# Exploring the limits of magnetic levitation: submicron particle separation and density profiling

**DOI:** 10.1088/2053-1591/add2d8

**Published:** 2025-05-09

**Authors:** Samantha Velazquez, Ali Akbar Ashkarran

**Affiliations:** 1 Department of Biology, University of Colorado Colorado Springs, Colorado Springs, CO, United States of America; 2 Department of Physics and Energy Science, University of Colorado Colorado Springs, Colorado Springs, CO, United States of America; 3 BioFrontiers Center, University of Colorado Colorado Springs, Colorado Springs, CO, United States of America

**Keywords:** magnetic levitation, density, brownian motion, particle size, biological applications

## Abstract

The size of particles plays a critical role in the time required to achieve stable levitation in magnetic levitation (MagLev) systems and significantly affects its practical applications, particularly in biological systems. There is a debate on the minimum size of the objects that can be levitated in a reasonable time period (e.g., <24 h). This challenge is particularly relevant in biological applications, where the small size of most biomolecules and the effects of Brownian motion pose significant concerns for biocompatibility. To address this issue, we have studied the lower limit of particle size possible for accurate density calculations using a ring MagLev system. Specifically, we examined commercially available polystyrene particles with known densities and identical physical properties, varying only in size from the microscale to the nanoscale. Our results demonstrate that although smaller particles (e.g., ∼200 nm) take considerably longer to achieve stable levitation compared to larger particles (e.g., 10 μm), they reach similar levitation heights, confirming the reliability of MagLev-based density measurements for submicron particles. Understanding the correlation of size and levitation time enables us to design/modify the MagLev experiments to minimize the exposure time of objects/particles to paramagnetic solutions, which is of key importance in the biomedical applications of MagLev systems.

## 1. Introduction

Magnetic levitation (MagLev) of diamagnetic objects or particles in a paramagnetic medium demonstrated great potential for concise, reproducible, and rapid density measurements [1–4]. This technique enabled researchers to address a wide range of challenges in materials science, biology, and physics [5–10]. In fact, density is one of the fundamental physical quantities of all materials. All substances have their exclusive density signature and quite a limited number of physical and chemical phenomenon experience variations in density [11–13]. Therefore, separations, sorting, and measurements based on density have attracted much attention within the scientific communities as an analytical technique [14–16].

Although, other techniques and approaches exist for density measurements of materials, such as, hydrometry [17], pycnometry [18], microchannel resonators [19], and so on [20], the mentioned techniques do not have the capacity to address the critical requirements like low cost, high accuracy, simplicity, and compactness at the same time. On the other hand, MagLev of diamagnetic materials in a paramagnetic medium is a robust technique for the measurement of density, which is low-cost, precise, fast, and straightforward [21]. MagLev was initially used for quality control of materials, probing the kinetics of chemical reactions, self-assembly of a variety of objects, food analysis, as well as separation of materials from mixtures [22–27]. However, in recent years, the applications of the MagLev technique have expanded significantly into the biological sciences, where it has proven to be a powerful tool for isolating biological molecules, studying cell behavior, and detecting disease biomarkers, ultimately demonstrating its potential to transform biomedical research and diagnostics [28–34].

Recently we have comprehensively reviewed the disease diagnostic capacity of the MagLev systems alone and when combined with other approaches [35]. For example, MagLev systems have been utilized to distinguish between healthy and cancerous cells, such as circulating tumor cells, primarily based on variations in their cellular densities [29]. Given that circulating tumor cells are indicative of metastasis, the MagLev system holds potential for real-time monitoring and risk assessment of secondary cancers. Yelinmez and co-workers showed that sickle cell disease can be diagnosed with MagLev [36]. Certain diseases exhibit characteristic density variations in specific cell types. For instance, MagLev technology has been demonstrated as a portable platform for on-site cell analysis, particularly for white blood cell cytometry [37, 38]. Tasoglu and colleagues developed a point-of-care MagLev device capable of distinguishing white blood cells from a heterogeneous blood sample, enhancing its functionality by integrating fluorescence imaging for clinical assays [39]. Furthermore, their smartphone-compatible miniaturized MagLev system enabled real-time, reproducible particle sorting in large-volume samples based on volumetric mass density, showcasing its potential for field-deployable diagnostics [40, 41].

The levitation time of particles in a MagLev system is highly dependent on size. When thermal fluctuations or Brownian motion exceed the combined gravitational and magnetic forces, smaller particles (∼1–2 μm) exhibit prolonged levitation times, often requiring several hours (2–24 h) for separation [42, 43]. For slightly larger objects (∼10 μm), separation occurs within a few hours, while particles around 50 μm stabilize in approximately 30 min [33]. This time-dependent behavior limits MagLev’s applicability in areas where rapid separation is essential, such as biological analyses and point-of-care diagnostics [44]. Experimental and theoretical studies have further indicated that conventional MagLev systems struggle to reliably measure the densities of objects smaller than ∼2 μm, as these particles tend to form diffuse clouds rather than discrete levitation layers, primarily due to the effects of Brownian motion [45].

Here, we explore the size limitations for reliable levitation and density-based analysis of particles using a ring MagLev system with various paramagnetic solutions. Moreover, we investigate the influence of particle size on levitation characteristics by probing the behavior of commercially available particles across a broad range of sizes. Our study provides insights into the factors affecting levitation time, stability, and accuracy in MagLev systems, particularly at smaller size scales.

## 2. Materials and methods

### 2.1. Materials

GdCl_3_, MnCl_2_, and DyCl_3_ were purchased from Sigma-Aldrich. Standard plain polystyrene beads of various sizes (i.e. 10 μm, 3 μm, 1 μm, 500 nm, 200 nm) and known density (*ρ* = 1.05 g cm^−3^) were provided by Polysciences (www.polysciences.com). Fluorescent polyethylene microparticles (see for example figure S1 of the supplementary information (SI)) with known densities and standard density solid glass particles were obtained for calibration of our MagLev system from Cospheric (www.cospheric.com) and American Density Materials (www.americandensitymaterials.com), respectively.

### 2.2. MagLev platform

**Figure 1.**
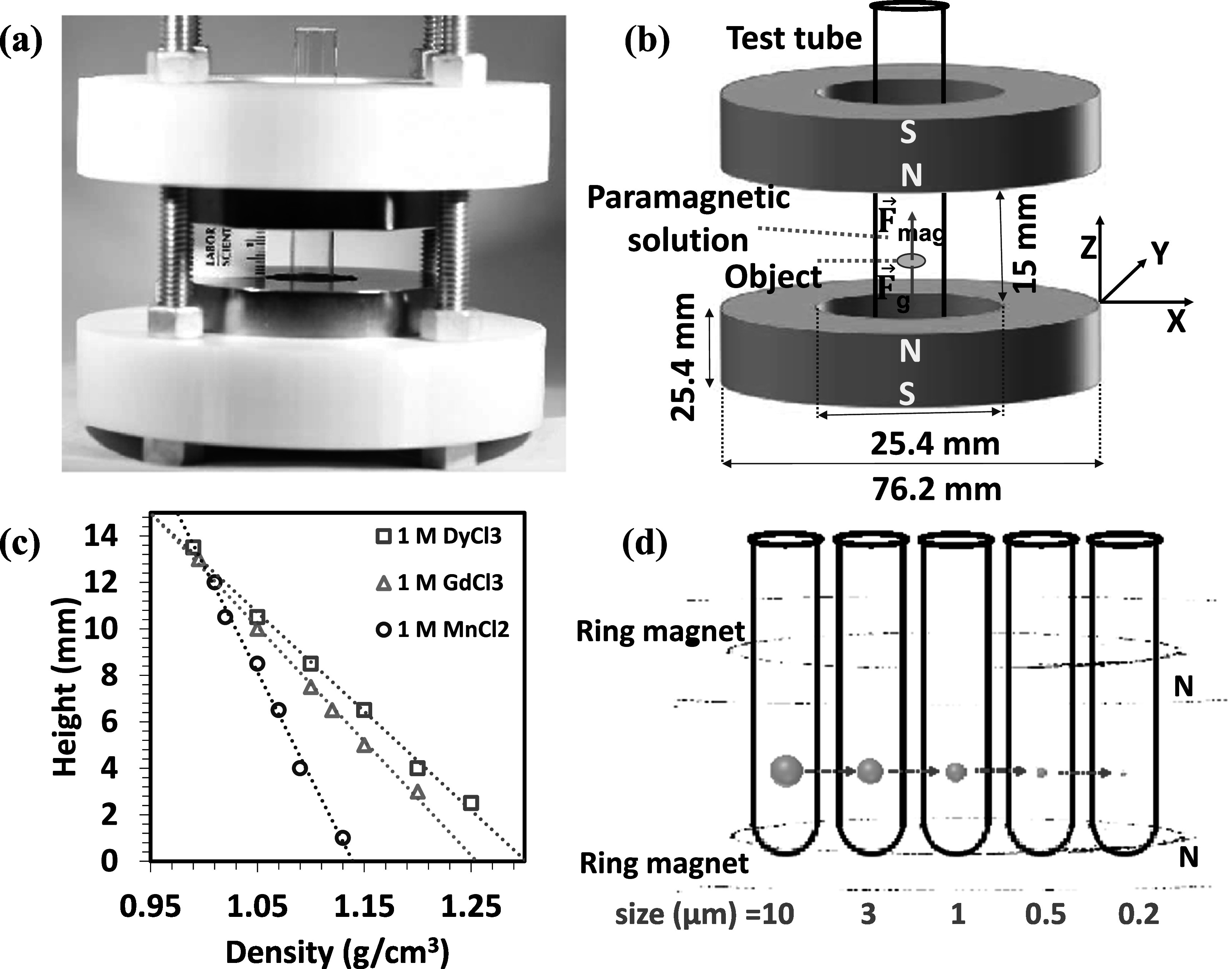
(a) Photograph, (b) schematic of the ring-MagLev configuration used in this research, (c) levitation heights of standard-density glass beads versus their densities showing calibration of the MagLev system and (d) scheme showing that density is not strictly a size-dependent function.

The ring MagLev system used in the present work is depicted in figure 1. The setup includes two blocks of neodymium ring-shaped permanent magnets (7.62 mm outer dimeter, 25.4 mm inner diameter, and 25.4 mm thickness), 1.5 cm distance of separation and N poles facing each other. Disposable glass test tubes with a diameter of 12 mm and length of 75 mm were used as levitation containers. To develop the high sensitivity MagLev system, we used two blocks of 4 × 2 × 1 inch cubic magnets with a separation distance of 7 cm and 90° rotation relative to the horizontal axis.

### 2.3. Characterization

Ring-shaped NdFeB permanent magnets (grade N42, Model NR022-4) and cubic shape magnets (grade N42, Model NB079) were provided from Applied Magnets (www.magnet4less.com) to develop the ring and high sensitivity Maglev systems. A Gauss meter (vector/magnitude Gauss meter model VGM, Alphalab) was used to measure the magnetic field strength between the magnets (∼0.4 T along the central axis between the magnets). Levitation profiles of the particles were recorded by a Nikon D750 digital camera containing a 105 mm Nikkor microlens.

## 3. Results and discussions

The minimum size of a particle that can be reliably levitated in a MagLev system can be estimated by considering the balance between thermal energy and the particle’s total energy. More specifically, a particle is expected to achieve a stable levitation height if its total energy exceeds the thermal energy at room temperature by at least one order of magnitude (see SI for calculations and derivations). It is previously estimated this lower size limit to be approximately r∼2 μm, assuming spherical particles, as the threshold for stable levitation and a reliable correlation between levitation height and density (see SI for more details) [45]. Polymer beads as test particles were used, with the smallest size being 1.5 μm, in a standard MagLev system consisting of two cubic permanent magnets with like poles facing each other. It is found that particles smaller than ∼2 μm in radius could not be consistently levitated or focused in such a configuration [45].

**Figure 2.**
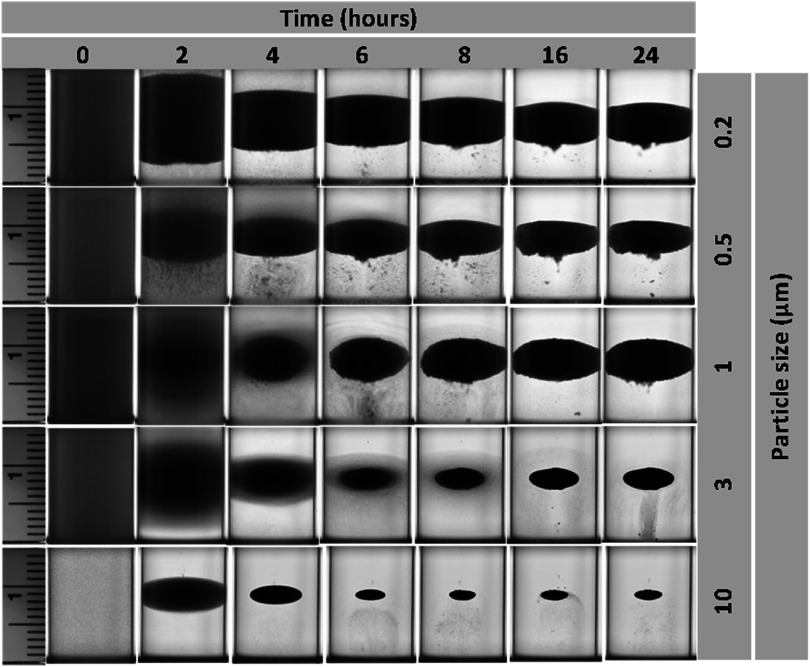
Levitation profiles of various sizes of polystyrene particles with known densities in 1 M concentration of MnCl_2_.

Figure 2 shows the levitation profile of various sized polystyrene particles with known densities in a 1 M concentration of MnCl_2,_ one of the most common paramagnetic solutions used in MagLev systems. A linear magnetic field gradient causes objects to move toward regions with lower magnetic field strength, effectively pushing them toward the center between the two magnets. The levitation profiles were monitored over 24 h (see movie S1 and S2 for levitation progress of 200 nm and 10 μm particles over time, respectively), which was particularly necessary for smaller particles; bigger particles take much less focusing time at final levitation height. The size range of 10 μm to 200 nm was selected based on the availability of commercially produced, identical particles (in terms of material, vendor, concentration, surface groups, dye, etc) to ensure consistency in the materials used throughout the study. It is worth noting that smaller commercial polystyrene particles (e.g., 40 nm) were available; however, they differed in color or surface functionalization and therefore were excluded from this study to maintain uniformity. The results revealed that density is not a strict function of particle size (figure 1(d)). Moreover, the results also demonstrate that although it can take significantly more time to separate/focus smaller particles (i.e., <1 micron), all particles show the same levitation height (approximately 9 mm).

We acknowledge the role of Brownian motion in influencing the behavior of smaller particles (e.g., <1 μm) within the MagLev system, causing temporary fluctuations before reaching equilibrium due to competing thermal, magnetic, and gravitational forces. These fluctuations are inversely proportional to particle size, with smaller particles exhibiting greater deviations before stabilizing at their final levitation height. Although real-time monitoring techniques, such as optical imaging, may have limitations in capturing rapid fluctuations at the microscale, these transient deviations do not significantly affect the time-averaged equilibrium position, thereby preserving the reliability and accuracy of the measurements.

**Figure 3.**
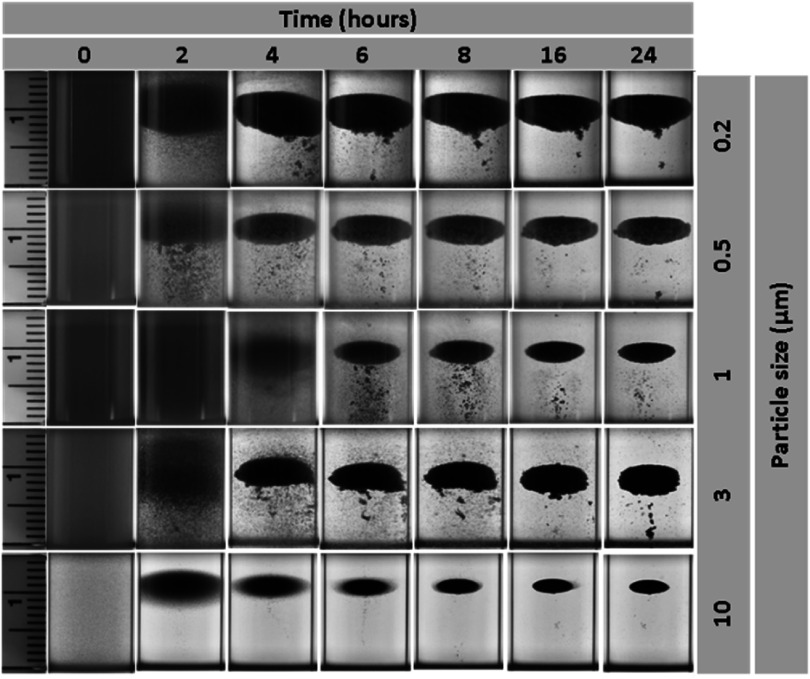
Levitation profiles of various sizes of polystyrene particles with known densities in 1 M concentration of GdCl_3_.

Figure 3 shows the levitation profiles of various sizes of polystyrene particles in a 1M concentration of GdCl_3_. The approximate center of the final clouds (i.e., after 24 h) appears at 10 ± 0.5 mm in the z direction, which suggests that particles with various sizes experience different equilibrium time, but their final levitation positions are almost the same in the z direction. Although the levitation profiles in GdCl_3_ are generally similar to the MnCl_2_ solution, i.e., the levitation process takes longer for smaller particles compared to bigger particles, the final levitation heights are a few millimeters higher compared with the MnCl_2_ solution. The observed variation in the levitation height is attributed, at least in large part, to the difference in magnetic susceptibility of these two paramagnetic solutions [46].

**Figure 4.**
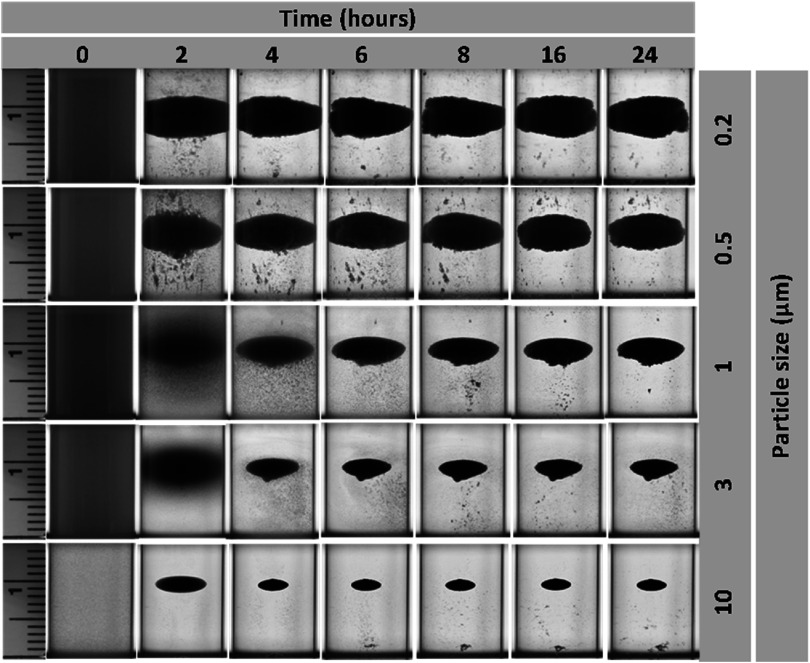
Levitation profiles of various sizes of polystyrene particles with known densities in 1 M concentration of DyCl_3_.

Interestingly, the levitation profiles of various sizes of polystyrene particles in a 1M concentration of DyCl_3_ exhibited trends similar to MnCl_2_ (figure 4). While levitation takes longer periods for smaller particles, the center of all final levitated clouds remains the same (approximately 10 mm). The observed differences among final levitation heights of the same polystyrene particles in different paramagnetic solutions are related to variations in their magnetic susceptibility. Table 1 summarizes the levitation heights of various sizes of polystyrene particles in 1M concentration of different paramagnetic solutions under two different experimental conditions (i.e., fully dispersed and non-dispersed).

**Table 1.** Summary of the levitation heights of polystyrene particles at different experimental conditions.

Size	Levitation height (mm)					
	Fully dispersed			Non-dispersed		
	GdCl_3_	MnCl_2_	DyCl_3_	GdCl_3_	MnCl_2_	DyCl_3_
10 μm	10.5	9	9.5	11	10	10
3 μm	10	8.5	9.5	11	10	10
1 μm	10	8.5	9	11.5	10	10
500 nm	10	8.5	9	11.5	10	10
200 nm	10	8.5	9	11	10	10

**Figure 5.**
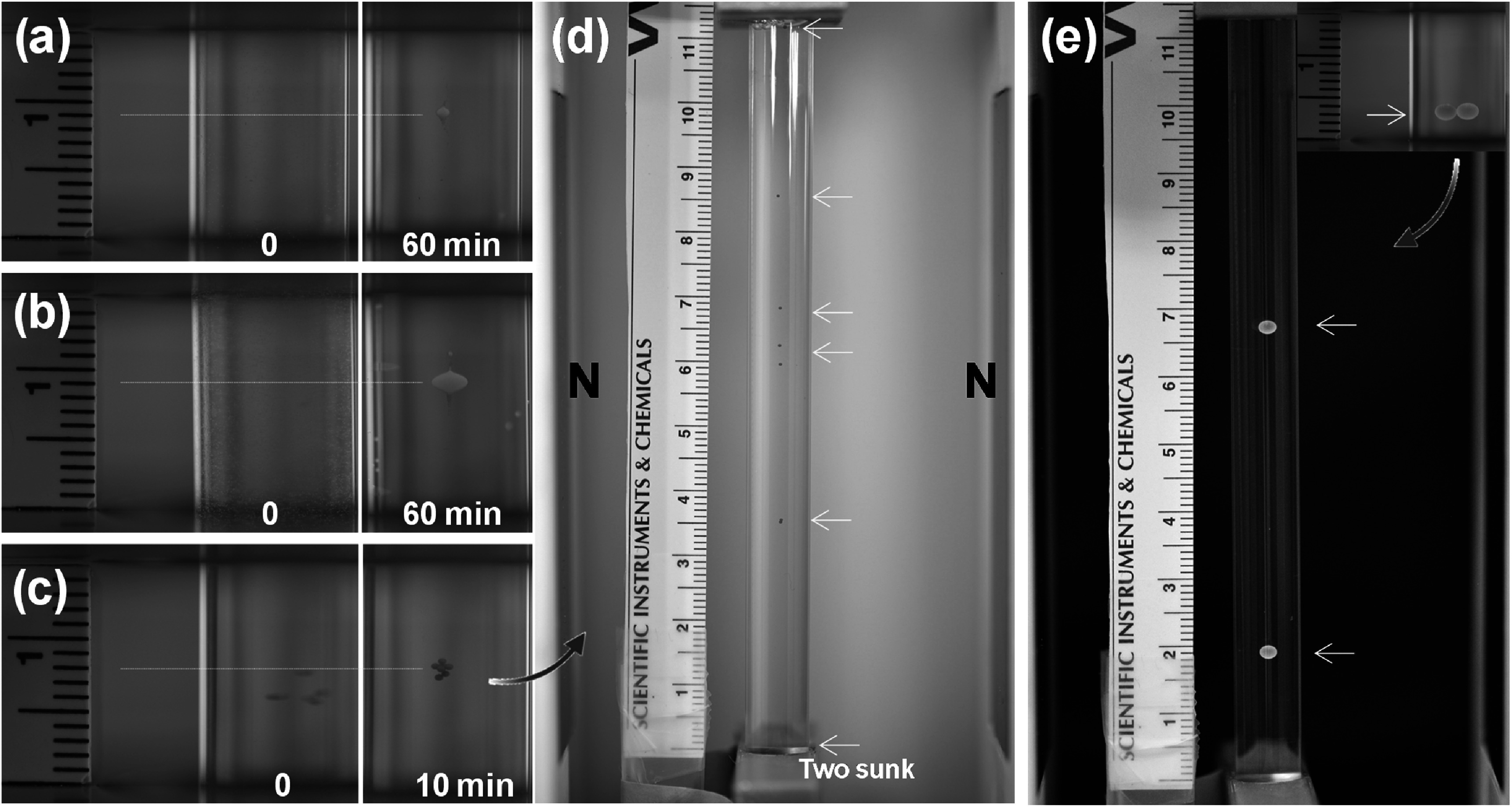
Levitation images of (a) 2 μl and (b) 15 μl of dispersed fluorescent polyethylene microspheres, with an average size of 50 μm, concentration of 100 mg ml^−1^ and a density of 1.055 g cm^−3^, in 1.0 M concentration of MnCl_2_ in a ring MagLev system at different times, (c) levitation images of 9 fluorescent polyethylene macroparticles, with an average size of 400 μm and a density of 1.065 g cm^−3^, in 1.0 M concentration of MnCl_2_ in a ring MagLev system at different times, (d) levitation profile of the same 9 fluorescent polyethylene macroparticles, with an average size of 400 μm and a density of 1.065 g cm^−3^ in 0.7M concentration of MnCl_2_ in a high sensitivity MagLev system (4 × 2 × 1 inch cubic magnets with a separation distance of 7 cm rotated 90° relative to the gravity direction), and (e) levitation profile of two polystyrene beads with a density of 1.05 g cm^−3^ and size of 1.9 mm in 0.25 M concentration of MnCl_2_ in ring Maglev system and 0.55 M concentration of MnCl_2_ in high sensitivity MagLev system.

Additionally, we tested the effect of particle quantity on levitation behavior and found that while the total amount of material influences the size of the levitated cloud, it does not significantly affect the final equilibrium height (figures 5(a)–(c)). The slight variations in levitation heights among particles primarily stem from minor density differences within the same batch, as no sample is perfectly uniform in density. This results in a small distribution of levitation heights, although the majority of particles settle at the same equilibrium position and share a consistent density value. However, these subtle density variations are not easily detectable due to the low sensitivity of the standard MagLev configuration. Even in bulk materials (e.g., various beads from the same batch) that appear to levitate at the same height in a standard ring MagLev system, significant differences in levitation height become apparent in high-sensitivity MagLev setups, highlighting the lower resolution of conventional MagLev systems (figures 5(d) and (e)).

**Figure 6.**
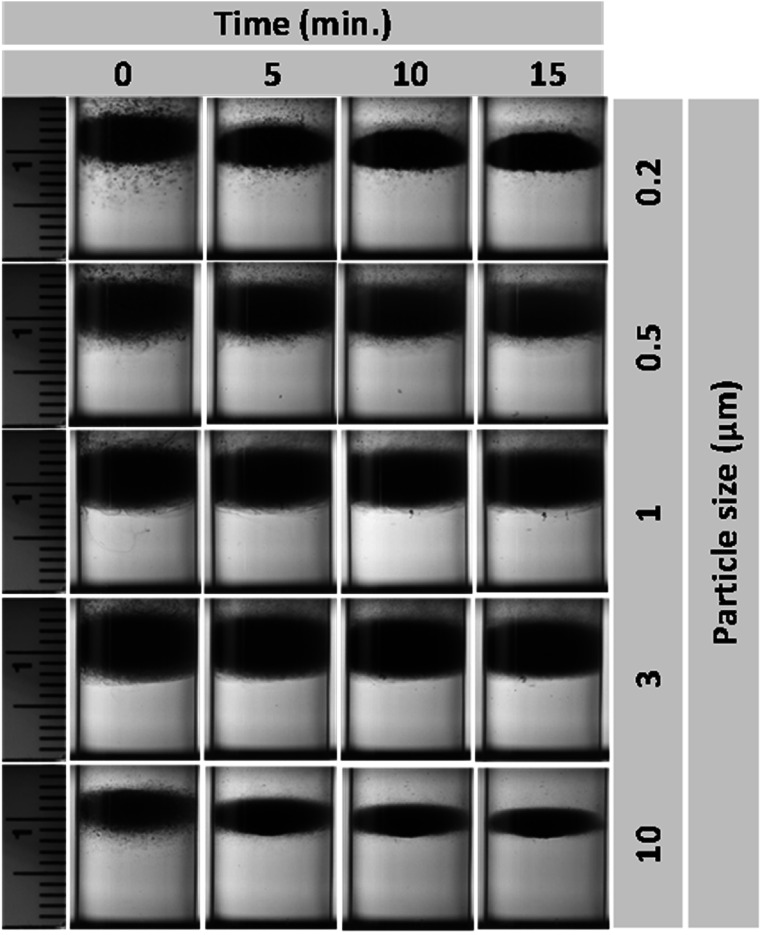
Levitation profiles of various sizes of polystyrene particles with known densities in 1 M concentration of MnCl_2_ without dispersion.

**Figure 7.**
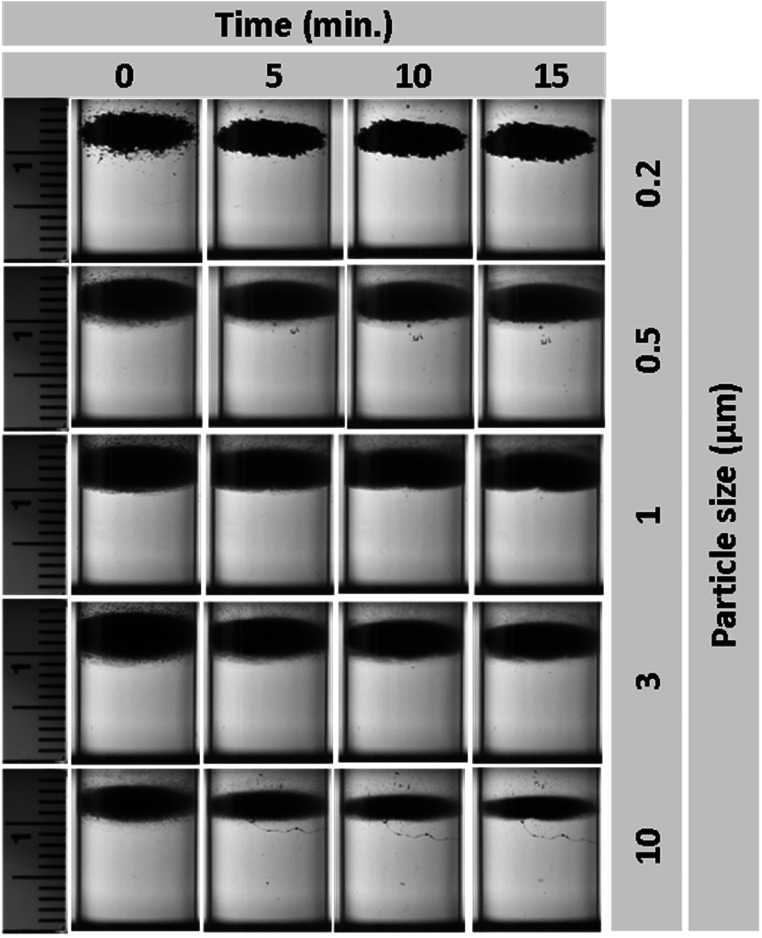
Levitation profiles of various sizes of polystyrene particles with known densities in 1 M concentration of GdCl_3_ without dispersion.

**Figure 8.**
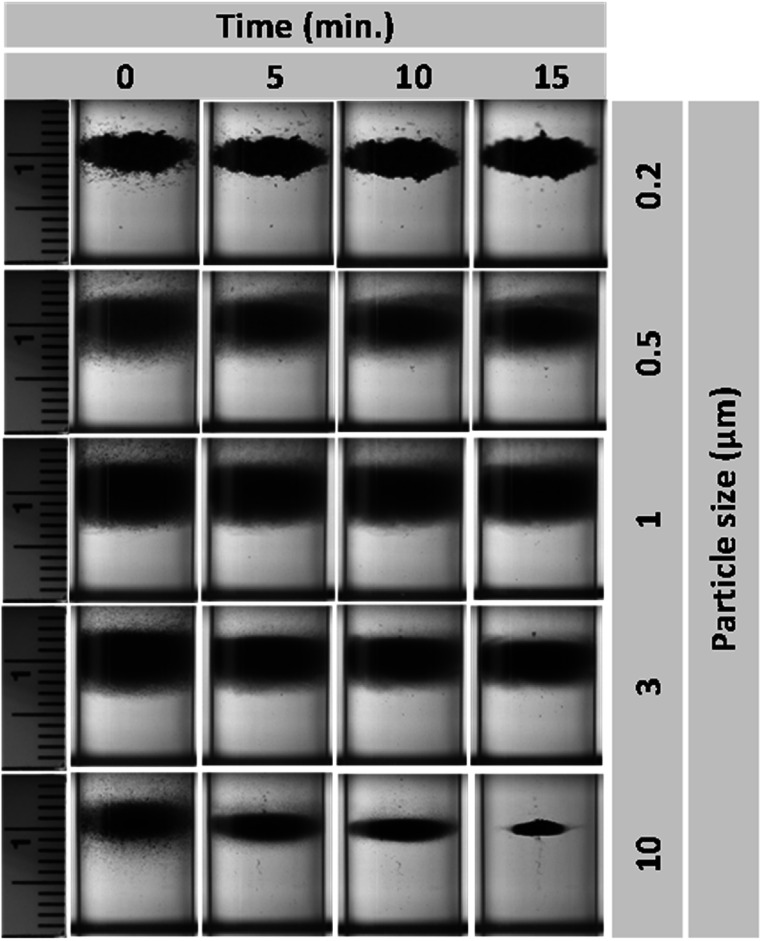
Levitation profiles of various sizes of polystyrene particles with known densities in 1 M concentration of DyCl_3_ without dispersion.

A different set of experiments was designed to study the effect of particle dispersity within the levitating medium. Figure 6 displays the levitation profiles of the same polystyrene particles of different sizes at 1 M concentration of MnCl_2_ but without being fully dispersed in the liquid. To evaluate the effect of particle dispersity on their levitation times, in the new set of experiments (see for example figures 6–8) we gently injected the same amount (volume and concentration) of polystyrene particles into the test tube located within the MagLev system (without any further pipetting or inducing turbulence) in 1 M concentration of three different paramagnetic solutions (see movie S3 and S4 for levitation progress of non-dispersed 200 nm and 10 μm particles in 1 M concentration of MnCl_2_, respectively). As is clear from the images in figure 6, there is no remarkable change in the geometry (e.g., shape and size) of the levitated cloud over time. Although there is slight variation (i.e., less than a millimeter) in the approximate center of the cloud, the overall shape and size of the initial levitated cloud remained the same.

This observation shows the crucial effect of experimental conditions for levitating micro and nanoscale particles/objects. The outcomes of the second set of experiments clearly indicate that non-dispersed (micro/nano) particles behave like bulk particles when they are not fully dispersed throughout the entire solution. This observation challenges the previous results/conclusions that MagLev cannot measure density at nanoscale (e.g., the density of biomolecules such as proteins, DNA, RNA, etc) [45]. Traditional density-based measurements in MagLev systems rely on the balance of magnetic and gravitational forces, resulting in a linear relationship between levitation height and material density. While this holds for bulk materials, nanoscale particles experience additional forces such as electrostatic interactions, thermal fluctuations, and van der Waals forces due to their high surface-to-volume ratio. At this scale, these interactions become significant and can no longer be ignored, as their magnitudes may be comparable to magnetic and gravitational forces [47].

Similarly, the levitation profiles of non-dispersed polystyrene particles of various size at 1 M concentrations of GdCl_3_ (figure 7) show no significant changes over time except ∼1 mm shift of the whole cloud downward. This may be due to the fact that we injected the particles from the top of the test tube into the MagLev solution. Therefore, the center of the cloud moves into its final equilibrium position (i.e. downward) over a few minutes.

Figure 8 demonstrates the levitation profiles of various sizes of polystyrene particles at 1 M concentrations of DyCl_3_ without dispersion. Similar trends were observed when using DyCl_3_ as the levitation medium. The downward shift of the levitated cloud after initial injection of the particles into the MagLev system is more remarkable for smaller particles, since the effect of Brownian motion is stronger for smaller particles (i.e., 200 nm) compared to larger particles (e.g., 10 μm). These results can be attributed to the fact that, in the absence of complete dispersion within the MagLev solution, the system contains particle aggregates rather than individual particles at the micrometer or nanometer scale. As a result, the effect of Brownian motion on a particle cluster differs significantly from its effect on fully dispersed individual particles. Consequently, if the particles are not evenly dispersed throughout the solution, Brownian motion is expected to have a reduced influence on their behavior.

**Figure 9.**
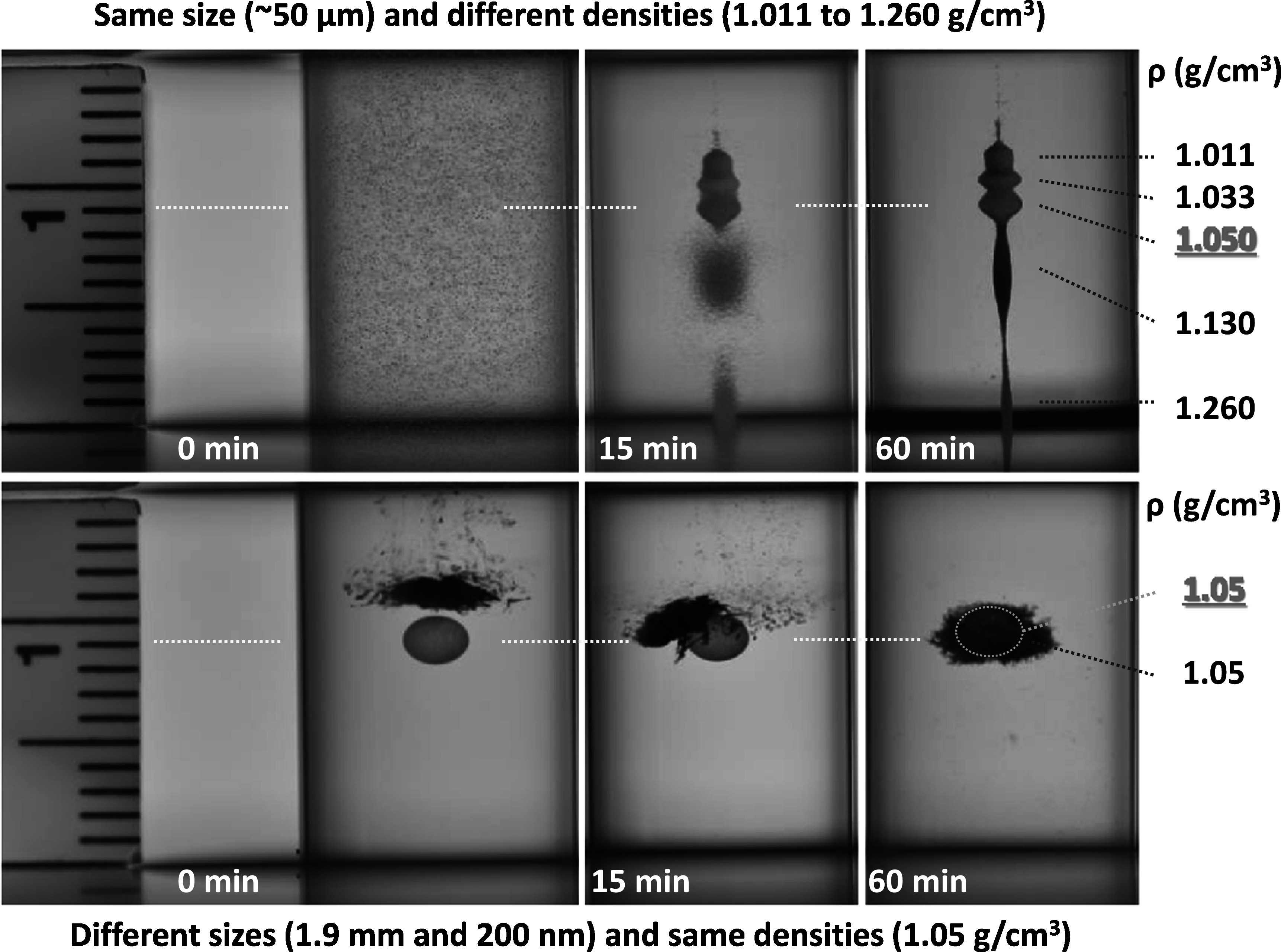
Levitation profiles of fluorescent polyethylene microspheres with the same size (∼50 μm) but different densities (top panel) and levitation characteristics of 1.9 mm and 200 nm polystyrene particles in 1 M concentration of MnCl_2_ (bottom panel) over time.

Figure 9 (top panel) shows the levitation profiles of fluorescent polyethylene microspheres with the same size (∼50 μm) but different densities in 1 M concentration of MnCl_2_. Given the size of the particles, focusing starts a few minutes after the particles are introduced into the MagLev system and is complete in less than an hour (see for example figure S1 and movies S5 to S7 for the entire levitation profiles over time in MnCl_2_, GdCl_3_, and DyCl_3_ solutions, respectively). As expected, particles of the same size but different densities exhibit different levitation heights. To confirm that density is not strictly a size-dependent function, we simultaneously levitated 1.9 mm and 200 nm polystyrene particles (bottom panel of figure 9). As is clear from the images, upon dropping the 1.9 mm bead into the MagLev solution (i.e. at t = 0), it goes to its final levitation position (i.e. 9 mm).

However, when the 200 nm particles are added into the MagLev system with the already levitating 1.9 mm particle, their primary levitation height is ∼11 mm, but they gradually (in less than an hour) move to their final levitation height (9 mm) and form a cloud around the 1.9 mm polystyrene bead (see figure S2 and movie S8 of SI). This observation confirms that density is not a strict function of the size of the particles/objects. Interestingly, not only are the final levitation heights of the 1.9 mm polystyrene bead and 200 nm particles with a density of 1.05 g cm^−3^ in the same experiment equal (bottom panel of figure 9), but in a different experiment, 50 μm fluorescent polyethylene microspheres with the same density (i.e., 1.05 g cm^−3^; the red microspheres in the top panel of figure 9) show exactly the same levitation height of millimeter- and nanometer-sized polystyrene particles.

It is important to note that experimental conditions also play a significant role in determining the levitation height of smaller size particles, particularly how samples are introduced into the MagLev system. In non-dispersed conditions, the sample behaves as a bulk mass rather than as individually separated particles. Since non-dispersed particles are not subjected to repeated pipetting, they do not fully disperse in the paramagnetic solution. As a result, they remain more localized rather than being evenly distributed throughout the medium. This reduced dispersion limits the influence of Brownian motion, leading to different levitation dynamics compared to fully dispersed samples. The faster equilibrium attainment of non-dispersed particles compared to fully dispersed ones may be attributed to reduced hydrodynamic drag, inter-particle interactions, and initial spatial distribution within the magnetic field. Aggregated particles experience lower drag due to their reduced surface-to-volume ratio, allowing for quicker movement. Additionally, weak inter-particle forces may facilitate cooperative settling, further accelerating their stabilization. In contrast, fully dispersed particles, influenced by Brownian motion and greater initial spatial variation, take longer to reach equilibrium. These factors highlight the role of dispersion state in levitation dynamics and suggest potential refinements for measurement methodologies.

The lower size limit of levitating objects/particles in MagLev systems remains an open question, further studies are needed to explore smaller particles (e.g., 1–100 nm). However, finding commercially available standard particles within this size range, with known densities and consistent physicochemical properties (except for size), is not straightforward. While MagLev has demonstrated its capability for density-based measurements, analysis, and separation of submicron particles/objects (e.g., most biomolecules) in addition to bulk objects, extending this technique to nanoscale objects presents unique challenges. These challenges stem from the concept of ‘effective density’, which becomes dominant at the nanoscale due to the interplay of additional forces and interactions between particles and the surrounding medium. In fact, at the bulk and microscale levels, the density of an object is primarily influenced by gravitational and magnetic forces, allowing for relatively straightforward density measurements using MagLev systems. However, at the lower end of the nanoscale range (e.g., 1–20 nm), other forces such as Brownian motion, electrostatic interactions, van der Waals forces, hydrogen bonding, and thermal fluctuations become more significant. These forces disrupt the traditional equilibrium between gravitational and magnetic forces, resulting in a shift from bulk density to what is known as effective density. This phenomenon occurs due to the high surface-to-volume ratio of nanoscale particles, which increases their interactions with the surrounding medium. For instance, nanoparticles are often coated with a hydration shell, where water molecules or other solvent molecules form a layer around the particle. This hydration shell alters the particle’s density by contributing to its overall mass without significantly increasing its volume. Consequently, the effective density measured by MagLev systems reflects both the intrinsic density of the nanoparticle and the contributions from the surrounding medium.

We have recently shown that while bulk materials exhibit stable and predictable levitation heights, nanoscale materials display more variability due to their effective density. For example, in the MagLev system, bulk silver particles with a density of approximately 10.5 g cm^−3^ cannot be levitated within the system’s dynamic range, but silver nanoparticles of the same composition can achieve stable levitation heights due to their altered effective density. This finding highlights the critical role of effective density at the nanoscale and suggests that MagLev systems must account for these changes to ensure accurate density measurements. Therefore, the shift from bulk density to effective density at the nanoscale is a key consideration for MagLev-based density measurements particularly for biomedical applications of MagLev systems, where minimizing exposure time to paramagnetic solutions is crucial to preserve the integrity of biological samples.

## 4. Conclusions

Previous reports have indicated that MagLev is incapable of accurately measuring the density of particles smaller than ∼2 microns due to the effects of Brownian motion. In this study, we used commercially available polystyrene particles of identical composition, with known densities and sizes ranging from the micro- to nanometer scale, to investigate the influence of Brownian motion on their levitation behavior. Our detailed analysis using a ring MagLev system revealed that particle density is not a size-dependent function. Furthermore, we observed that particles of different sizes exhibit the same levitation height, although smaller particles take significantly longer to reach their equilibrium position. Additionally, we demonstrated that non-dispersed particles achieve their levitation height much more rapidly (within a few minutes) compared to fully dispersed particles. These findings suggest that while materials of the same density, but different sizes ultimately reach identical levitation heights, their time to equilibrium varies based on particle size and dispersion state.

## Data Availability

All data that support the findings of this study are included within the article (and any supplementary files).
